# Awareness and Prescribing Practices for Doxycycline Post‐Exposure Prophylaxis (doxyPEP) Among Healthcare Providers Delivering Sexual Health Care in Australia: A Cross‐Sectional Survey

**DOI:** 10.5694/mja2.70329

**Published:** 2026-11-03

**Authors:** Haein Lee, Fabian Y. S. Kong, Ei T. Aung, Vincent J. Cornelisse, Teralynn Ludwick, Christopher K. Fairley, Benjamin Riley, Phyu Mon Latt, Eric P. F. Chow

**Affiliations:** ^1^ School of Translational Medicine, Faculty of Medicine, Nursing and Health Sciences Monash University Melbourne Victoria Australia; ^2^ Melbourne Sexual Health Centre Bayside Health Carlton Victoria Australia; ^3^ Melbourne School of Public Health The University of Melbourne Melbourne Victoria Australia; ^4^ Mid North Coast Local Health District Port Macquarie New South Wales Australia; ^5^ Kirby Institute UNSW Sydney Sydney New South Wales Australia; ^6^ Australasian Society for HIV, Viral Hepatitis and Sexual Health Medicine Sydney New South Wales Australia

**Keywords:** drug resistance, public health, sexually transmitted diseases

## Abstract

We surveyed 215 healthcare providers delivering sexual health care in Australia about doxycycline post‐exposure prophylaxis (doxyPEP) for sexually transmitted infection prevention. Awareness was high (190/215, 88.4%) and half of those aware (94/190, 49.5%) had prescribed doxyPEP in the past 6 months. Among those who had not prescribed doxyPEP in the past 6 months, concern about antimicrobial resistance was the most frequently reported reason (85/121, 70.2%).

## 1 Introduction

Doxycycline post‐exposure prophylaxis (doxyPEP), consisting of 200 mg of doxycycline taken within 72 h of sexual exposure, is an effective strategy for reducing the incidence of sexually transmitted infections (STIs) [1]. In 2024, the Australasian Society for Human Immunodeficiency Virus (HIV), Viral Hepatitis and Sexual Health Medicine (ASHM) published a consensus statement recommending doxyPEP primarily for men who have sex with men (MSM) at risk of syphilis [2]. More recently, in July 2026, the World Health Organization published its first guidelines on doxyPEP [3]. Australian MSM are already using doxyPEP, often with non‐recommended regimens and unsupervised sourcing [4], but data on clinician prescribing practices remain limited. We surveyed healthcare providers delivering sexual health care across Australia to describe their awareness, knowledge and prescribing practices regarding doxyPEP.

## 2 Methods

We conducted an anonymous, cross‐sectional online survey of healthcare providers delivering sexual health care in Australia from 27 September 2024 to 30 April 2025 using Qualtrics software (Provo, US). Eligible participants were providing HIV and sexual health care in Australia and included general practitioners (GPs), sexual health physicians, infectious diseases physicians and nurse practitioners. Participants were recruited through professional organisations, social media and snowball sampling. The survey collected data on awareness and knowledge of doxyPEP, prescribing practices in the past 6 months, willingness to prescribe across clinical scenarios and concerns about antimicrobial resistance (AMR). Survey methodology, including the Checklist for Reporting Of Survey Studies (CROSS), is reported in the Supporting Information (Supporting Information, Sections S1 and S2). This project was reviewed and approved by the Alfred Hospital Ethics Committee (Project Number: 498/24) and registered with Monash University Human Research Ethics Committee (Project Number: 46130).

## 3 Results

A total of 215 participants completed the survey and were included in the final analyses. The median age was 46.4 years (interquartile range: 36–56 years). Over half (116/215, 54.0%) were cisgender women, and 46.5% (100/215) were based in Victoria (Table 1). GPs comprised the largest group (100/215, 46.5%), including 36 s100 prescribers (medical practitioners [general practitioners, nurse practitioners and other community‐based medical practitioners] who are authorised to prescribe HIV and hepatitis B medications under the Australian Government's Highly Specialised Drugs Program), followed by sexual health physicians (59/215, 27.4%) (Table S1). Most participants (179/215, 83.3%) reported a specific interest in sexual health medicine, and 99/215 (46.0%) reported that sexual health consultations accounted for up to 25% of their workload.

**TABLE 1:** Characteristics and sexual health workload of 215 survey participants.

Characteristics	Statistics
Age (years), median (interquartile range)	46.4 (36–56)
Gender, *n* (%)	
Cisgender woman	116 (54.0%)
Cisgender man	88 (40.9%)
Transgender, non‐binary, or other	11 (5.1%)
State/territory, *n* (%)	
Victoria	100 (46.5%)
New South Wales	48 (22.3%)
Other states/territories	67 (31.2%)
Professional group, *n* (%)	
General practitioners (including s100 prescribers)^a^	100 (46.5%)
Sexual health physicians	59 (27.4%)
Infectious diseases physicians	34 (15.8%)
Other (e.g., nurse practitioners, public health)	22 (10.2%)
Sexual health workload, *n* (%)	
Specific interest in sexual health medicine	179 (83.3%)
Consultations related to sexual health (0%–25%)	99 (46.0%)
Consultations related to sexual health (76%–100%)	79 (36.7%)

Abbreviation: s100, medical practitioners (general practitioners, nurse practitioners and other community‐based medical practitioners) who are authorised to prescribe HIV and hepatitis B medications under the Australian Government's Highly Specialised Drugs Program.

^a^ Includes 36 human immunodeficiency virus (HIV) s100 prescribers authorised to manage highly specialised drugs.

Overall, 190/215 (88.4%) participants had heard of doxyPEP (Figure 1A, Table S2), whereas only 123/215 (57.2%) had read the ASHM doxyPEP consensus statement (Table S3); readership was lowest among GPs (41/100, 41.0%). Among those aware, 73.7% (140/190) correctly identified the recommended dosing regimen. Awareness of doxyPEP was universal among sexual health physicians (59/59, 100%) and lower among GPs (78/100, 78.0%).

**FIGURE 1:**
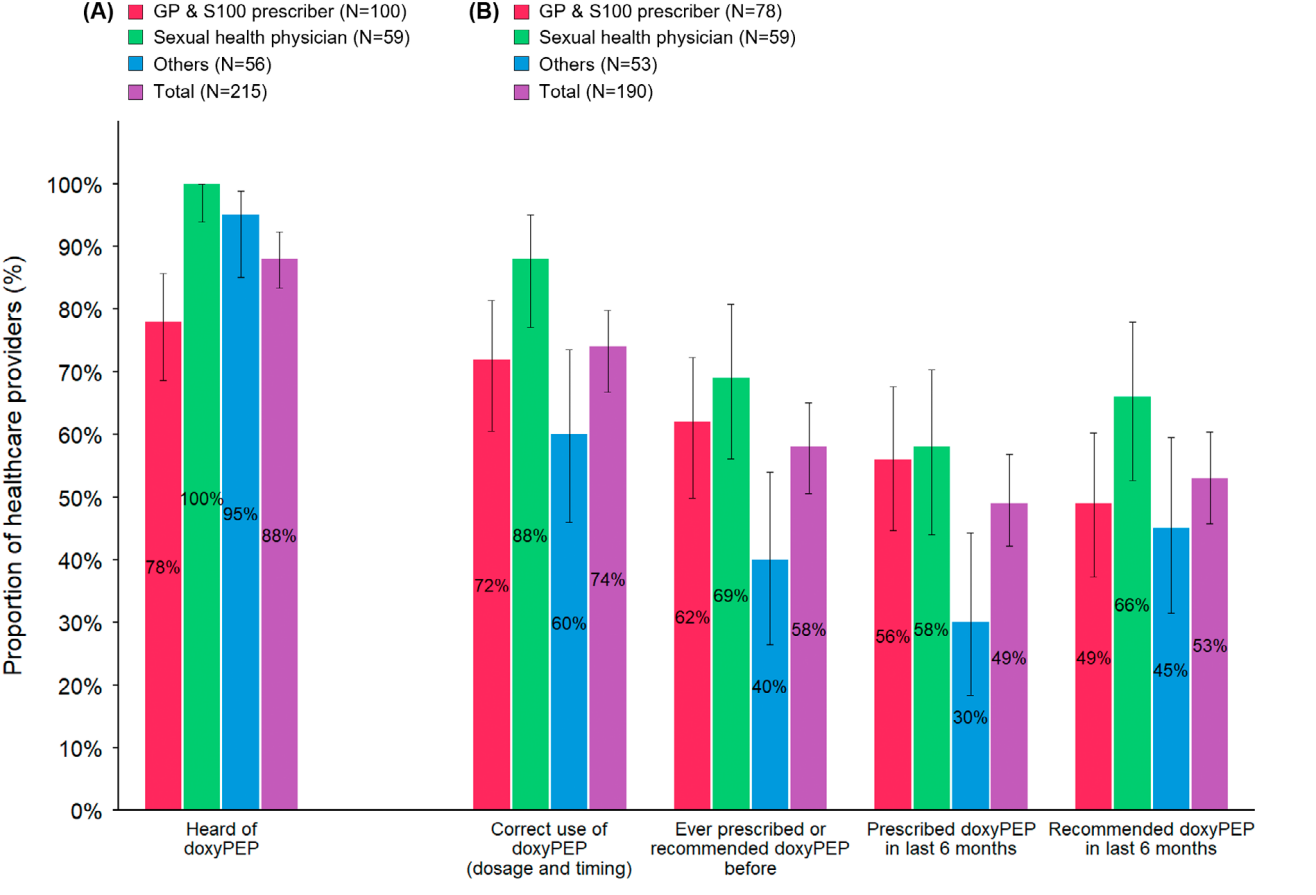
Awareness and knowledge of doxycycline post‐exposure prophylaxis (doxyPEP) among healthcare providers, by professional group: (A) awareness among 215 participants and (B) knowledge and prescribing or recommending among the 190 participants aware of doxyPEP. GP, general practitioner; *N*, total number of participants in each group; doxyPEP, doxycycline post‐exposure prophylaxis; s100 prescriber, medical practitioners (general practitioners, nurse practitioners and other community‐based medical practitioners) who are authorised to prescribe human immunodeficiency virus and hepatitis B medications under the Australian Government's Highly Specialised Drugs Program. (A) Percentage of participants in each group who have heard of doxyPEP. (B) Among those aware (*N* = 190), percentages show responses to follow‐up knowledge questions within each group. 95% confidence interval error bars are shown on the figure. Confidence intervals were calculated using the binomial exact method.

Among those aware of doxyPEP, 49.5% (94/190) had prescribed doxyPEP in the past 6 months (Figure 1B, Table S4). Willingness to prescribe was highest for MSM with two or more recent bacterial STI diagnoses (161/215, 74.9%) or recent syphilis (160/215, 74.4%), and lower for low‐risk MSM requesting the medication (98/215, 45.6%) (Table S5). Although the ASHM consensus statement does not address doxyPEP for Indigenous women, half of the participants (109/215, 50.7%) reported that they would be willing to prescribe doxyPEP to Indigenous women at risk of syphilis. Among those who had not prescribed doxyPEP in the past 6 months, concern about AMR was the most frequently cited reason (85/121, 70.2%), followed by insufficient knowledge of doxyPEP (49/121, 40.5%) (Table S6). Almost all (207/215, 96.3%) expressed concern about AMR, and 48.8% (105/215) were moderately or very concerned that doxyPEP could increase AMR in commensal organisms (Table S7). Among doxyPEP prescribers, 61.7% (58/94) reported always discussing AMR, and 48.9% (46/94) reported always discussing potential changes to the microbiome with clients (Table S8).

## 4 Discussion

Our survey found high awareness of doxyPEP among healthcare providers delivering sexual health care in Australia, but prescribing in the past 6 months was reported by about half of the aware providers. International surveys from Italy, Brazil and the United States have also examined healthcare providers' attitudes and prescribing practices regarding doxyPEP, although measures and study populations differed [5, 6, 7].

Several factors may contribute to this pattern. Nearly half of the participants reported a low volume of sexual health consultations, meaning many may have had limited opportunity to identify eligible clients rather than a reluctance to prescribe. Where clinicians had not prescribed, concern about AMR was the most reported reason, which aligns with evidence that doxyPEP is associated with increased tetracycline resistance in *Neisseria gonorrhoeae* [8, 9]. However, AMR concern was equally high among providers who did prescribe, suggesting it does not fully account for variation in practice. The lower willingness to prescribe for populations where trial evidence is lacking, such as Indigenous women at risk of syphilis, likely reflects genuine uncertainty about the evidence base rather than resistance to the intervention itself. Recent Australian modelling found that all doxyPEP strategies for MSM were cost‐saving, with a syphilis‐diagnosis‐based targeting strategy achieving the highest benefit–cost ratio while minimising the increase in tetracycline‐resistant gonorrhoea [10]. However, extending prescribing to populations for which trial evidence is lacking will require additional data.

This study has several limitations. The sample was recruited through sexual health networks, and most participants reported a pre‐existing interest in sexual health, so awareness and prescribing are likely overestimated relative to the broader GP workforce. Participants were disproportionately from Victoria. Self‐reported prescribing is subject to recall bias.

These findings underscore the need for wider dissemination of existing doxyPEP guidance, particularly among GPs, and for ongoing research into the long‐term impact of doxyPEP on antimicrobial resistance in Australia.

## Author Contributions

Conceptualisation: E.P.F.C. Data curation: H.L., F.Y.S.K., E.T.A., V.J.C., B.R., C.K.F., E.P.F.C. Formal analysis: H.L., P.M.L., E.P.F.C. Funding acquisition: E.P.F.C. Methodology: H.L., F.Y.S.K., E.T.A., T.L., V.J.C., E.P.F.C. Project administration: H.L., P.M.L., E.P.F.C. Resources: P.M.L., E.P.F.C. Software: P.M.L., E.P.F.C. Supervision: P.M.L., E.P.F.C. Validation: P.M.L., E.P.F.C. Visualisation: P.M.L. Writing – original draft: H.L. Writing – review and editing: H.L., F.Y.S.K., E.T.A., V.J.C., T.L., C.K.F., B.R., P.M.L., E.P.F.C.

## Funding

E.P.F.C. and C.K.F. are each supported by a National Health and Medical Research Council (NHMRC) Leadership Investigator Grant (GNT2033299 and GNT1172900, respectively). P.M.L. and E.T.A. are supported by E.P.F.C.'s NHMRC Leadership Investigator Grant (GNT2033299). F.Y.S.K. is supported by an NHMRC Emerging Leadership Investigator Grant (GNT2033078). The funding sources had no role in the survey's design, implementation or analysis.

## Disclosure

Not commissioned; externally peer reviewed.

## Conflicts of Interest

The authors declare no conflicts of interest.

## Supporting information


**Supporting Information:** mja270329‐sup‐0001‐Supinfo.pdf. **Section S1:** Survey development, recruitment, data quality and statistical analysis.
**Section S2:** Consensus‐Based Checklist for Reporting of Survey Studies (CROSS).
**Table S1:** Demographic and professional characteristics of the 215 survey participants.
**Table S2:** Awareness of ASHM consensus statement and of doxyPEP among 215 participants and identification of the recommended doxyPEP regimen among 190 participants aware of doxyPEP, by professional group.
**Table S3:** Familiarity with and interest in reading the ASHM consensus statement among 215 survey participants.
**Table S4:** (A) prescribing and recommending practices of doxyPEP among 190 participants who were aware of doxyPEP and (B) reported client benefits and adverse effects among 94 participants who prescribed doxyPEP in the past 6 months.
**Table S5:** Proportion of healthcare participants delivering sexual healthcare who would prescribe or recommend doxyPEP to clients in various hypothetical clinical situations (*N* = 215).
**Table S6:** Reasons for (a) prescribing/recommending doxyPEP among 94 participants who had prescribed it in the past 6 months and (b) not prescribing/recommending doxyPEP among 121 participants who had not prescribed doxyPEP in the past 6 months for STI prevention.
**Table S7:** Anticipated effect of doxyPEP on STIs in Australian communities and concerns about antimicrobial resistance among 215 survey participants.
**Table S8:** Views on doxyPEP effectiveness (*N* = 215) and discussion of antimicrobial resistance and microbiome changes with clients (*N* = 94 prescribers).

## Data Availability

All data relevant to the study are included in the article or are available as Supporting Information.
