# Supplementary material for: Awareness and Prescribing Practices for Doxycycline Post‐Exposure Prophylaxis (doxyPEP) Among Healthcare Providers Delivering Sexual Health Care in Australia: A Cross‐Sectional Survey
Source: Med J Aust. 2026 Nov 3;224(11):e70329. doi: 10.5694/mja2.70329 (PMC13730544; doi:10.5694/mja2.70329)
Supplement: Supplementary file 1 [file MJA2-224-0-s001.pdf]

MJA
The Medical Journal of Australia

Supporting Information

Supplementary material

**This appendix was part of the submitted manuscript and has been peer reviewed.
It is posted as supplied by the authors.**

Appendix to: Lee H, Kong FYS, Aung ET, et al. Awareness and prescribing practices for doxycycline post-exposure prophylaxis (doxyPEP) among health care providers delivering sexual health care in Australia: a cross-sectional survey. *Med J Aust* 2026; doi: 10.5694/mja2.70329.

SUPPLEMENTARY MATERIALS

Section S1. Survey development, recruitment, data quality and statistical analysis

Study Design and Study Population

We conducted an online, anonymous, cross-sectional survey to explore the views and prescribing practices of health care providers delivering sexual health care regarding doxyPEP in Australia. The survey was administered using Qualtrics software (Provo, USA) between 27 September 2024 and 30 April 2025. The questionnaire was co-designed by epidemiologists, sexual health physicians, pharmacists and social scientists.

To be eligible, participants were required to: (1) be currently living in Australia, (2) be aged 18 years or older, and (3) be currently practising in Australia as general practitioners including s100 prescribers (i.e. medical practitioners who are authorised to prescribe HIV and hepatitis B medications under the Australian Government's Highly Specialised Drugs Program), specialists in HIV, sexual health, and/or infectious diseases or nurse practitioners who provided HIV or sexual health care.

Participants were recruited through targeted outreach to key organisations, including the Australasian Society for HIV, Viral Hepatitis and Sexual Health Medicine (ASHM), The Royal Australian College of General Practitioners (RACGP), Victorian HIV and Hepatitis Integrated Training and Learning (VHITTAL) program, The Australasian Chapter of Sexual Health Medicine (AChSHM), The Australasian Society for Infectious Diseases (ASID), Centre for Excellence in Rural Sexual Health (CERSH), Victorian Primary Care Practice-based Research Network (VicRen). The survey was also promoted via social media (Facebook, Instagram, X, and LinkedIn), email invitations to general practice clinics across Australia, and snowball sampling.

A brief introduction at the beginning of the survey provided participants with an overview of the study. Informed consent was obtained electronically, where participants could indicate consent by selecting the "I consent to participate" button or decline by selecting "I do not consent to participate." All participants were required to confirm their eligibility and consent before commencing the survey.

Data collection and measures

The survey comprised four main sections: (1) demographic characteristics (e.g. age, gender, years in practice, geographical location of workplace, medical specialty, training, experience in providing sexual health care to LGBTIQA+ clients); (2) awareness of doxyPEP (e.g. whether they had heard of doxyPEP, how it is used, and which infections it is effective in preventing); (3) prescribing practices of doxyPEP for STI prevention (e.g. whether they had ever prescribed or recommended doxyPEP and associated reasons, discussions around antimicrobial resistance (AMR) or impacts on the microbiome when prescribing doxyPEP, and how likely they would be to prescribe or recommend doxyPEP for the 11 different hypothetical clinical scenarios); (4) views on doxyPEP (e.g. their beliefs whether doxyPEP would reduce STIs) in Australia and any concerns.

Statistical analyses

In this analysis, we included participants who met predefined data quality criteria. To ensure the quality of responses, we implemented several fraud-detection measures in accordance with Qualtrics' recommendations. We excluded responses with a high fraud score (≥30), a low reCAPTCHA score (<0.5), those that failed the bot check or did not answer the attention-check question correctly.

Descriptive statistics (e.g. median, interquartile range, frequency and proportion) were used to report the study variables. Subgroup analyses were performed by specialty (GPs, including those accredited as s100 prescribers; sexual health physicians; and others, including nurse practitioners).

All statistical analyses were performed in Stata/MP (version 19.5, College Station, TX, USA), and all figures were generated in R (version 4.3.2; R Foundation for Statistical Computing, Vienna, Austria).

Section S2. Consensus-Based Checklist for Reporting of Survey Studies (CROSS)

Section/topic	Item	Item description	Reported on page #
**Title and abstract**			
Title and abstract	1a	State the word "survey" along with a commonly used term in title or abstract to introduce the study's design.	Research Letter, p. 1
	1b	Provide an informative summary in the abstract, covering background, objectives, methods, findings/results, interpretation/discussion, and conclusions.	Research Letter, p. 1
**Introduction**			
Background	2	Provide a background about the rationale of study, what has been previously done, and why this survey is needed.	Research Letter, p. 1
Purpose/aim	3	Identify specific purposes, aims, goals, or objectives of the study.	Research Letter, p. 1
**Methods**			
Study design	4	Specify the study design in the methods section with a commonly used term (e.g., cross-sectional or longitudinal).	Research Letter, p. 1; Supp. File, p. 1
Data collection methods	5a	Describe the questionnaire (e.g., number of sections, number of questions, number and names of instruments used).	4-5
	5b	Describe all questionnaire instruments that were used in the survey to measure particular concepts. Report target population, reported validity and reliability information, scoring/classification procedure, and reference links (if any).	NA
	5c	Provide information on pretesting of the questionnaire, if performed (in the article or in an online supplement). Report the method of pretesting, number of times questionnaire was pre-tested, number and demographics of participants used for pretesting, and the level of similarity of demographics between pre-testing participants and sample population.	NA
	5d	Questionnaire if possible, should be fully provided (in the article, or as appendices or as an online supplement).	Supplementary File
Sample characteristics	6a	Describe the study population (i.e., background, locations, eligibility criteria for participant inclusion in survey, exclusion criteria).	Research Letter, p. 2; Supp. File, p. 1, 2
	6b	Describe the sampling techniques used (e.g., single stage or multistage sampling, simple random sampling, stratified sampling, cluster sampling, convenience sampling). Specify the locations of sample participants whenever clustered sampling was applied.	Supp. File, p. 1–2
	6c	Provide information on sample size, along with details of sample size calculation.	-
	6d	Describe how representative the sample is of the study population (or target population if possible), particularly for population-based surveys.	Research Letter, P. 4
Survey administration	7a	Provide information on modes of questionnaire administration, including the type and number of contacts, the location where the survey was conducted (e.g., outpatient room or by use of online tools, such as SurveyMonkey).	Supp. File, p. 1
	7b	Provide information of survey's time frame, such as periods of recruitment, exposure, and follow-up days.	Research Letter, p. 1; Supp. File, p. 1
	7c	Provide information on the entry process:<br>–>For non-web-based surveys, provide approaches to minimize human error in data entry.<br>–>For web-based surveys, provide approaches to prevent "multiple participation" of participants.	Supp. File, p. 2
Study preparation	8	Describe any preparation process before conducting the survey (e.g., interviewers' training process, advertising the survey).	Supp. File, p. 1
Ethical considerations	9a	Provide information on ethical approval for the survey if obtained, including informed consent, institutional review board [IRB] approval,	Supp. File, p. 2

		Helsinki declaration, and good clinical practice [GCP] declaration (as appropriate).	
	9b	Provide information about survey anonymity and confidentiality and describe what mechanisms were used to protect unauthorized access.	Supp. File, p. 2
Statistical analysis	10a	Describe statistical methods and analytical approach. Report the statistical software that was used for data analysis.	Supp. File, p. 2
	10b	Report any modification of variables used in the analysis, along with reference (if available).	NA
	10c	Report details about how missing data was handled. Include rate of missing items, missing data mechanism (i.e., missing completely at random [MCAR], missing at random [MAR] or missing not at random [MNAR]) and methods used to deal with missing data (e.g., multiple imputation).	NA
	10d	State how non-response error was addressed.	NA
	10e	For longitudinal surveys, state how loss to follow-up was addressed.	NA
	10f	Indicate whether any methods such as weighting of items or propensity scores have been used to adjust for non-representativeness of the sample.	NA
	10g	Describe any sensitivity analysis conducted.	NA
**Results**			
Respondent characteristics	11a	Report numbers of individuals at each stage of the study. Consider using a flow diagram, if possible.	NA
	11b	Provide reasons for non-participation at each stage, if possible.	NA
	11c	Report response rate, present the definition of response rate or the formula used to calculate response rate.	NA
	11d	Provide information to define how unique visitors are determined. Report number of unique visitors along with relevant proportions (e.g., view proportion, participation proportion, completion proportion).	NA
Descriptive results	12	Provide characteristics of study participants, as well as information on potential confounders and assessed outcomes.	Research Letter, p. 2, 5
Main findings	13a	Give unadjusted estimates and, if applicable, confounder-adjusted estimates along with 95% confidence intervals and p-values.	Research Letter, p.6
	13b	For multivariable analysis, provide information on the model building process, model fit statistics, and model assumptions (as appropriate).	NA
	13c	Provide details about any sensitivity analysis performed. If there are considerable amount of missing data, report sensitivity analyses comparing the results of complete cases with that of the imputed dataset (if possible).	NA
**Discussion**			
Limitations	14	Discuss the limitations of the study, considering sources of potential biases and imprecisions, such as non-representativeness of sample, study design, important uncontrolled confounders.	Research Letter, p. 3
Interpretations	15	Give a cautious overall interpretation of results, based on potential biases and imprecisions and suggest areas for future research.	Research Letter, p. 3
Generalizability	16	Discuss the external validity of the results.	Research Letter, p. 3
**Other sections**			
Role of funding source	17	State whether any funding organization has had any roles in the survey's design, implementation, and analysis.	Yes
Conflict of interest	18	Declare any potential conflict of interest.	Yes
Acknowledgements	19	Provide names of organizations/persons that are acknowledged along with their contribution to the research.	Yes

Table S1. Demographic and professional characteristics of the 215 survey participants

Characteristics	Statistics
Age in years, median (IQR)	46.4 (36 to 56)
**Gender, *n* (%)**	
Cisgender man	88 (40.9%)
Cisgender woman	116 (54.0%)
Transgender, non-binary or other	11 (5.1%)
**State/Territory, n (%)**	
Victoria	100 (46.5%)
New South Wales	48 (22.3%)
Western Australia	24 (11.2%)
Queensland	19 (8.8%)
South Australia	15 (7.0%)
Australian Capital Territory	5 (2.3%)
Northern Territory	2 (0.9%)
Tasmania	2 (0.9%)
**Remoteness area (n), %**	
Major cities	163 (75.8%)
Inner regional	25 (11.6%)
Outer regional, remote, and very remote	27 (12.6%)
**Speciality (n), %**	
GP[1]	64 (29.8%)
GP and s100 prescriber*	36 (16.7%)
ID physician[2]	34 (15.8%)
Nurse practitioner[3]	15 (7.0%)
Sexual health physician[4]	59 (27.4%)
Public health physician	3 (1.4%)
Microbiologists[5]	2 (0.9%)
Others[6]	2 (0.9%)
**Years of practice (median, IQR)**	**12 (5-25)**
**Interest in sexual health medicine (n), %**	
Yes	179 (83.3%)
No	35 (16.3%)
Missing/Unknown	1 (0.5%)
**Proportion of consultations related to sexual health issues, concerns, or treatments for sexually transmitted infections (n), %**	
0-25%	99 (46.0%)
26-50%	24 (11.2%)
51-75%	13 (6.0%)
76-100%	79 (36.7%)

n, number; IQR, interquartile range; GP, general practitioner; ID, infectious diseases

1 includes 1 GP registrar working in a sexual health clinic
2 includes 1 general medicine, ID, microbiology and 1 ID and clinical microbiology
3 includes 1 clinical nurse specialist HIV/sexual health
4 includes 1 sexual health advanced trainee, junior doctor working in a sexual health clinic
5 includes 1 clinical microbiologist, 1 medical microbiologist
6 includes 1 resident medical officer, 1 emergency medicine
* medical practitioners (general practitioners, nurse practitioners and other community-based medical practitioners) who are authorised to prescribe HIV and hepatitis B medications under the Australian Government Department of Health and Aged Care's Section 100 (s100).

Table S2. Awareness of ASHM consensus statement and of doxyPEP among 215 participants and identification of the recommended doxyPEP regimen among 190 participants aware of doxyPEP, by professional group

	GP & S100 prescriber	Sexual health physician	Others	Total
Awareness of ASHM consensus statement, n/N (%)				
Aware and read	41/100 (41.0%)	51/59 (86.4%)	31/56 (55.4%)	123/215 (57.2%)
Aware but not read	24/100 (24.0%)	7/59 (11.9%)	10/56 (17.9%)	41/215 (19.1%)
Not aware	35/100 (35.0%)	1/59 (1.7%)	15/56 (26.8%)	51/215 (23.7%)
Heard of doxyPEP, n/N (%)				
Yes	78/100 (78.0%)	59/59 (100.0%)	53/56(94.6%)	190/215 (88.4%)
No	22/100 (22.0%)	0/59 (0.0%)	3/56 (5.4%)	25/215 (11.6%)
Identifying the recommended doxyPEP regimen, n/N (%)				
Yes	56/78 (71.8%)	52/59 (88.1%)	32/53 (60.4%)	140/190 (73.7%)
No	22/78 (28.2%)	7/59 (11.9%)	21/53 (39.6%)	50/190 (26.3%)

Table S3. Familiarity with and interest in reading the ASHM consensus statement among 215 survey participants

**ASHM awareness (N=215)**	**(n), %**
Aware and read	123 (57.2%)
Aware but not read	41 (19.1%)
Not aware	51 (23.7%)
**Aware of statement and interested in reading (N=41)**	**(n), %**
Yes	34 (82.9%)
No	1 (2.4%)
Unsure	5 (12.2%)
Missing/Unknown	1 (2.4%)
**Unaware of the statement and interested in reading (N=51)**	**(n), %**
Yes	46 (90.2%)
No	1 (2.0%)
Unsure	4 (7.8%)

ASHM, Australasian Society for HIV, Viral Hepatitis and Sexual Health Medicine; ASHM consensus statement, a consensus statement published by ASHM following a national roundtable discussion on the use of doxyPEP in Australia; statement refers to ASHM consensus statement.

GP, general practitioner; n, number; N, total number of participants in each group, ASHM, the Australasian Society for HIV, Viral Hepatitis and Sexual Health Medicine; doxyPEP, doxycycline post-exposure prophylaxis; percentages indicate the proportion of participants selecting each option.

Table S4. (A) prescribing and recommending practices of doxyPEP among 190 participants who were aware of doxyPEP and (B) reported client benefits and adverse effects among 94 participants who prescribed doxyPEP in the past 6 months

(A) Prescribing and recommending practices of doxyPEP among 190 participants who were aware of doxyPEP				
	**GP & S100 prescriber**	**Sexual health physician**	**Others**	**Total**
Prescribed/recommended doxyPEP, n/N (%)	48/78 (61.5%)	41/59 (69.5%)	21/53 (39.6%)	110/190 (57.9%)
Prescribed doxyPEP in the past 6 months, n/N (%)	44/78 (56.4%)	34/59 (57.6%)	16/53 (30.2%)	94/190 (49.5%)
Recommended doxyPEP in the past 6 months, n/N (%)	38/78 (48.7%)	39/59 (66.1%)	24/53 (45.3%)	101/190 (53.2%)
**(B) Reported client benefits and adverse effects among 94 participants who prescribed doxyPEP in the past 6 months**				
**Client reporting benefits**				
Yes				31 (33.0%)
No				19 (20.2%)
I do not know				44 (46.8%)
**Client reporting adverse effects**				
Yes				10 (10.6%)
No				71 (75.5%)
I do not know				13 (13.8%)

GP, general practitioner; s100 prescriber, medical practitioners (general practitioners, nurse practitioners and other community-based medical practitioners) who are authorised to prescribe HIV and hepatitis B medications under the Australian Government's Highly Specialised Drugs Program; doxyPEP, doxycycline post-exposure prophylaxis; n, number; N, total number of participants in each group; percentages indicate the proportion of participants selecting each option.

Table S5. Proportion of healthcare participants delivering sexual healthcare who would prescribe or recommend doxyPEP to clients in various hypothetical clinical situations. (N=215)

Client scenario	Not at all n (%)	Unlikely n (%)	Likely n (%)	Always n (%)
Gay/bisexual men with recent syphilis	12 (5.6%)	43 (20.0%)	136 (63.3%)	24 (11.2%)
Gay/bisexual men with ≥ 2 recent bacterial STIs	9 (4.2%)	45 (20.9%)	132 (61.4%)	29 (13.5%)
Men with male and female partners	16 (7.4%)	77 (35.8%)	113 (52.6%)	9 (4.2%)
Gay/bisexual men not using condoms	12 (5.6%)	69 (32.1%)	124 (57.7%)	10 (4.7%)
Gay/bisexual men on HIV PrEP	12 (5.6%)	67 (31.2%)	120 (55.8%)	16 (7.4%)
Gay/bisexual men presenting for HIV PEP	9 (4.2%)	69 (32.1%)	115 (53.5%)	22 (10.2%)
Gay/bisexual men using chemsex	13 (6.0%)	63 (29.3%)	121 (56.3%)	18 (8.4%)
Immunocompromised clients	14 (6.5%)	85 (39.5%)	106 (49.3%)	10 (4.7%)
Sex workers	13 (6.0%)	92 (42.8%)	99 (46.0%)	11 (5.1%)
Low-risk gay/bisexual men requesting doxyPEP	26 (12.1%)	91 (42.3%)	87 (40.5%)	11 (5.1%)
Indigenous women at syphilis risk	23 (10.7%)	83 (38.6%)	97 (45.1%)	12 (5.6%)

STI, sexually transmitted infection; doxyPEP, doxycycline post-exposure prophylaxis; MSM, men who have sex with men; HIV, human immunodeficiency virus; HIV PEP, HIV post-exposure prophylaxis; HIV PrEP, HIV pre-exposure prophylaxis; chemsex, "the voluntary intake of psychoactive and non-psychoactive drugs in the context of recreational settings to facilitate and/or to enhance sexual intercourse mostly among men who have sex with other men (MSM)".

Table S6. Reasons for (a) prescribing/recommending doxyPEP among 94 participants who had prescribed it in the past 6 months and (b) not prescribing/recommending doxyPEP among 121 participants who had not prescribed doxyPEP in the past 6 months for STI prevention

(a). Reason(s)* for prescribing or recommending doxyPEP (N=94)	n (%)
There is enough evidence of effectiveness for me to prescribe doxyPEP	80 (85.1%)
It can reduce the ongoing transmission of STIs and reduce STI prevalence	74 (78.7%)
Clients ask me to prescribe it	74 (78.7%)
There is enough evidence of safety for me to prescribe doxyPEP	73 (77.7%)
I understand doxyPEP well	54 (57.4%)
Other clinician(s) in my practice are also prescribing doxyPEP to their client	44 (46.8%)
Clients using it have less anxiety during sex	32 (34.0%)
**(b). Reason(s) for not prescribing or recommending doxyPEP (N =121)**	**n (%)**
I am worried that doxyPEP may increase antibiotic resistance in some infections	85 (70.2%)
I do not know enough about doxyPEP and need to read more about it	49 (40.5%)
Not enough evidence of effectiveness for me to prescribe doxyPEP	16 (13.2%)
Other clinician(s) in my practice are not prescribing doxyPEP to their client(s)	14 (11.6%)
Not enough evidence of safety for me to prescribe doxyPEP	12 (9.9%)
I am worried that my client(s) would not take doxyPEP correctly	12 (9.9%)
I do not have enough time to adequately discuss doxyPEP with my client(s)	9 (7.4%)
I generally don't know my client's risk of STIs	8 (6.6%)
I read the resources about doxyPEP, but they are difficult to understand or interpret	2 (1.7%)
I am not confident in providing sexual health consultations and discussing sexual health with my clients	0 (0.0%)

*Participants can select more than one option; doxyPEP, doxycycline post-exposure prophylaxis; n, number; N, total number of participants in each group; STI, sexually transmitted infection; percentages indicate the proportion of participants selecting each reason.
Panel (a) shows reasons among 94 participants who prescribed doxyPEP in the past six months; panel (b) shows reasons among 121 participants who did not.

Table S7. Anticipated effect of doxyPEP on STIs in Australian communities and concerns about antimicrobial resistance among 215 survey participants

STI reduction/AMR concern (N=215)	n (%)
Anticipated reduction in STIs in Australian communities due to doxyPEP, n (%)	
Very unlikely	7 (3.3%)
Unlikely	17 (7.9%)
I do not know	69 (32.1%)
Likely	98 (45.6%)
Very likely	24 (11.2%)
Concerns around AMR due to doxyPEP, n (%)	
Not at all concerned	8 (3.7%)
Slightly concerned	76 (35.3%)
Moderately concerned	70 (32.6%)
Very concerned	61 (28.4%)
Concerns that doxyPEP will increase AMR in commensal organisms, n (%)	
Not at all concerned	21 (9.8%)
Slightly concerned	89 (41.4%)
Moderately concerned	66 (30.7%)
Very concerned	39 (18.1%)

STI, sexually transmitted infection; doxyPEP, doxycycline post-exposure prophylaxis; AMR, antimicrobial resistance.

Table S8. Views on doxyPEP effectiveness (N=215) and discussion of antimicrobial resistance and microbiome changes with clients (N=94 prescribers)

Scenario item	n/N (%)
**DoxyPEP should be considered for the prevention of STIs in all gay, bisexual and queer men**	
Strongly disagree	38/215 (17.7%)
Disagree	62/215 (28.8%)
Neither disagree nor agree	39/215 (18.1%)
Agree	55/215 (25.6%)
Strongly agree	21/215 (9.8%)
**DoxyPEP is effective in preventing chlamydia in gay, bisexual and queer men**	
Strongly disagree	2/215 (0.9%)
Disagree	6/215 (2.8%)
Neither disagree nor agree	31/215 (14.4%)
Agree	117/215 (54.4%)
Strongly agree	59/215 (27.4%)
**DoxyPEP is effective in preventing syphilis in gay, bisexual and queer men**	
Strongly disagree	2/215 (0.9%)
Disagree	6/215 (2.8%)
Neither disagree nor agree	19/215 (8.8%)
Agree	118/215 (54.9%)
Strongly agree	70/215 (32.6%)
**DoxyPEP is effective in preventing gonorrhoea in gay, bisexual and queer men**	
Strongly disagree	22/215 (10.2%)
Disagree	72/215 (33.5%)
Neither disagree nor agree	69/215 (32.1%)
Agree	46/215 (21.4%)
Strongly agree	6/215 (2.8%)
**Discussion question items**	**n/N (%)**
**Discussing AMR with patients when prescribing doxyPEP**	
Never	1/94 (1.1%)
Rarely	5/94 (5.3%)
Sometimes	12/94 (12.8%)
Often	18/94 (19.2%)
Always	58/94 (61.7%)
**Discussing changes to the microbiome**	
Never	5/94 (5.3%)
Rarely	7/94 (7.4%)
Sometimes	18/94 (19.1%)
Often	18/94 (19.1%)
Always	46/94 (48.9%)

doxyPEP, doxycycline post-exposure prophylaxis; n, number; N, total number of participants in each group - For scenario item, N=215, For discussion items, N=94 (those who prescribed doxyPEP in the past 6 months); AMR, antimicrobial resistance.
